# Supplementary material for: The Mitochondrial Genomes of Siboglinum plumosum and Oligobrachia dogieli (Annelida: Siboglinidae) and Their Phylogenetic Analysis
Source: Genes (Basel). 2024 Jan 7;15(1):77. doi: 10.3390/genes15010077 (PMC10815697; doi:10.3390/genes15010077)
Supplement: Supplementary file 1 [file genes-15-00077-s001.zip › genes-2757406-supplementary.pdf]

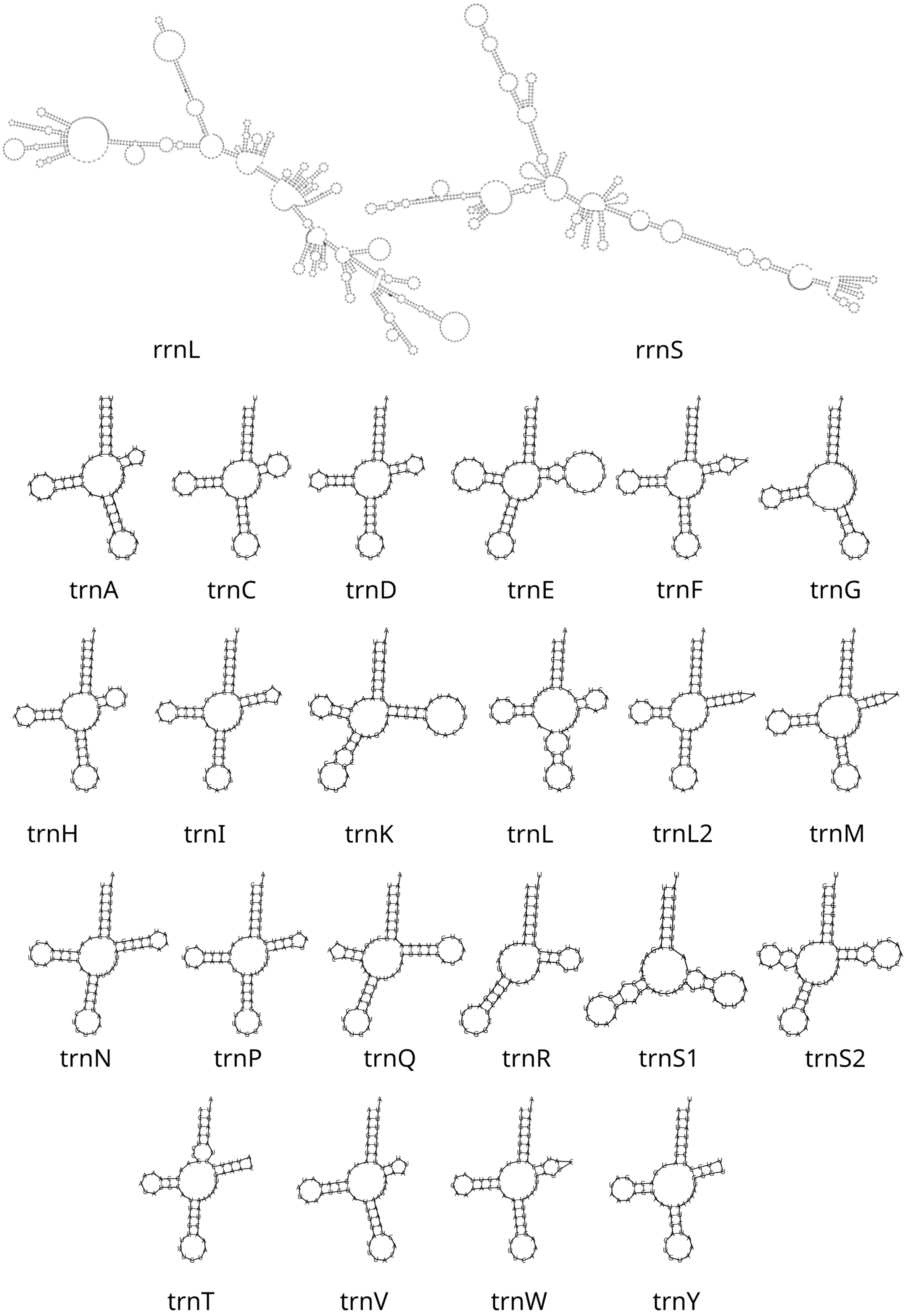

Figure S1. The putative secondary structures of ribosomal and transfer RNAs of *S. plumosum* mitogenome.

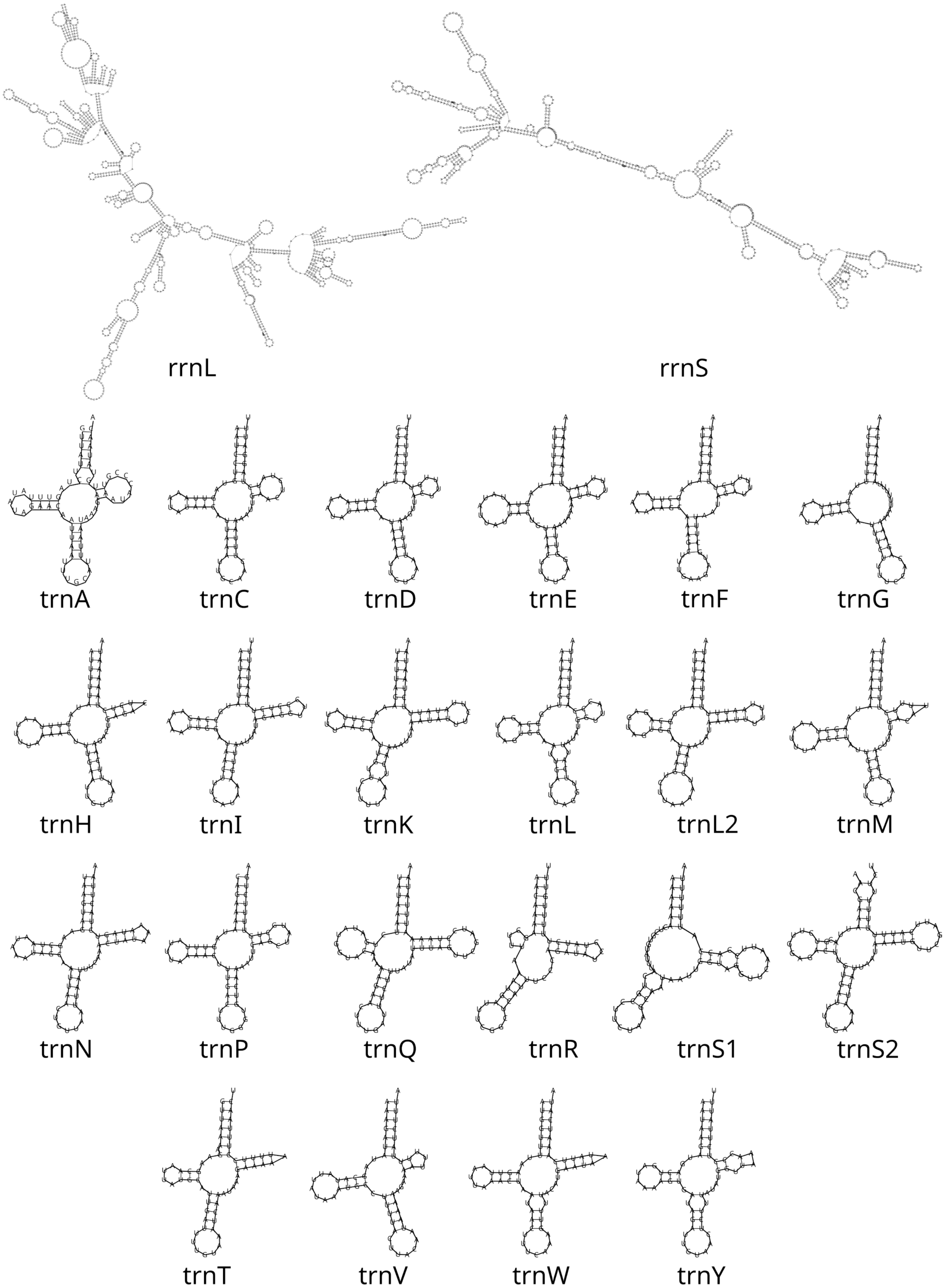

Figure S2. The putative secondary structures of ribosomal and transfer RNAs of *O. dogieli* mitogenome.

Table S1. NCBI Accession numbers of COX1 and 16S rRNA genes of siboglinid species used in single-gene phylogenetic analyses.

NCBI Accession	Full name	COI	16S
FJ480356.1	Bobmarleya gadensis isolate 1	yes	no
FJ480357.1	Bobmarleya gadensis isolate AT615G3	yes	no
NC_026857	Galathealinum brachiosum	yes	yes
U74066.1	Galathealinum brachiosum	yes	no
FJ480376.1	Lamellisabella denticulata isolate AT622-G4	yes	no
FJ480377.1	Lamellisabella denticulata isolate Lam1	yes	no
FJ480388.1	Lamellisabella sp. SBJ-2009 isolate Lam2	yes	no
OR804078.1	Oligobrachia dogieli	yes	yes
MK673146.1	Oligobrachia haakonmosbiensis isolate AD3	yes	no
MK673147.1	Oligobrachia haakonmosbiensis isolate AD4	yes	no
OP541392.1	Oligobrachia haakonmosbiensis isolate BC2_5, Kvalsund	yes	no
MH393883.1	Oligobrachia haakonmosbiensis isolate BS1	yes	no
MH619672.1	Oligobrachia haakonmosbiensis voucher 22	yes	no
MH619665.1	Oligobrachia haakonmosbiensis voucher 23	yes	no
MH619669.1	Oligobrachia haakonmosbiensis voucher 26	yes	no
MH619666.1	Oligobrachia haakonmosbiensis voucher 28	yes	no
MH619671.1	Oligobrachia haakonmosbiensis voucher 33	yes	no
MH619686.1	Oligobrachia haakonmosbiensis voucher VI-023-Nyegga	yes	no
MH619683.1	Oligobrachia haakonmosbiensis voucher VI-135-HMMV	yes	no
MH619685.1	Oligobrachia haakonmosbiensis voucher VI-143-1-HMMV	yes	no
MH619661.1	Oligobrachia sp. 1078-2	yes	no
MH619674.1	Oligobrachia sp. 1125-9	yes	no
MH619680.1	Oligobrachia sp. 1154-6	yes	no
MW598476.1	Oligobrachia sp. ovs3 voucher ZMMU-WS14754	yes	no
MH619687.1	Oligobrachia sp. P11-LaptevSea	yes	no
MK673155.1	Oligobrachia sp. Vestnesa isolate AD13	yes	no
MK673149.1	Oligobrachia sp. Vestnesa isolate AD6	yes	no
MK673151.1	Oligobrachia sp. Vestnesa isolate AD9	yes	no
OP541394.1	Oligobrachia webbi isolate voucher	yes	no
FJ480393.1	Polybrachia sp. 1 SBJ-2009 isolate BJG3	yes	no
FJ480389.1	Polybrachia sp. 2 SBJ-2009 isolate Poly1	yes	no
FJ480391.1	Polybrachia sp. 3 SBJ-2009 isolate Poly2	yes	no
FJ480392.1	Polybrachia sp. 3 SBJ-2009 isolate Poly3	yes	no
AF315037.1	Polybrachia sp. HRV-2001	no	yes
KJ789169.1	Siboglinum ekmani	yes	yes
KF444429.1	Siboglinum ekmani	yes	no
OP541395.1	Siboglinum ekmani isolate St1 G1, Nordfjord	yes	no
OP541396.1	Siboglinum ekmani isolate St1 G2_1, Nordfjord	yes	no
OP541397.1	Siboglinum ekmani isolate St1 G2_2, Nordfjord	yes	no
NC_026833.1	Siboglinum fiordicum	yes	yes
OP541390.1	Siboglinum fiordicum isolate BC1_2, Kvalsund	yes	no
OP541391.1	Siboglinum fiordicum isolate BC2_1, Kvalsund	yes	no
MK673145.1	Siboglinum fiordicum isolate NewAS8	yes	no
MK673144.1	Siboglinum fiordicum isolate NewAS9	yes	no
OP541393.1	Siboglinum fiordicum isolate VVG_8, Nordfjord	yes	no
OR551480.1	Siboglinum plumosum	yes	yes
FJ480399.1	Siboglinum poseidoni isolate CapG2	yes	no
FJ480398.1	Siboglinum poseidoni isolate CapG40	yes	no
FJ483547.1	Spirobrachia cf. grandis SJ-2009	yes	no

KJ789171.1	Spirobrachia sp. YL-2014	yes	yes
FJ480371.1	Spirobrachia tripeira isolate AT622-G2	yes	no
FJ480373.1	Spirobrachia tripeira isolate AT622-G5	yes	no
FJ480374.1	Spirobrachia tripeira isolate AT622-G6	yes	no
FJ480375.1	Spirobrachia tripeira isolate AT622-G7	yes	no
MT108937.1	Osedax rubiplumus	yes	yes
